# Multiscale Modeling Approach for the Aldol Addition Reaction in Multicompartment Micelle-Based Nanoreactor

**DOI:** 10.1021/acs.jpcb.3c05858

**Published:** 2023-11-13

**Authors:** Jinwon Cho, Marcus Weck, Sungu Hwang, Seung Soon Jang

**Affiliations:** † Computational NanoBio Technology Laboratory, School of Materials Science and Engineering, Georgia Institute of Technology, 771 Ferst Drive NW, Atlanta, Georgia 30332-0245, United States; ‡ Molecular Design Institute and Department of Chemistry, New York University, New York, New York 10003, United States; § Department of Nanomechatronics Engineering, Pusan National University, Miryang 50463, Korea

## Abstract

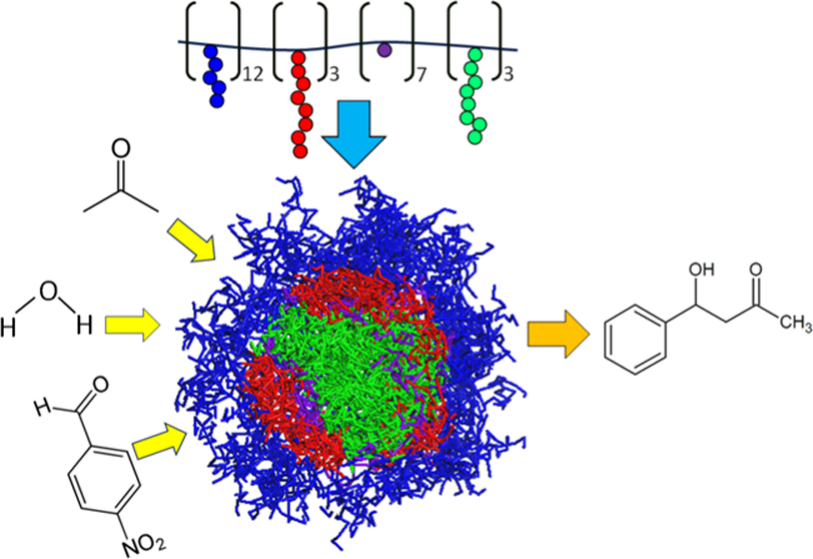

Water has emerged as a versatile solvent for organic chemistry in recent years due to its abundance, low cost, and environmental friendliness. However, one of the most important reactions, the aldol reaction, in the presence of excess water exhibits low yields and poor enantioselectivities. In this regard, we have employed a multiscale modeling approach to investigate the aldol addition reaction catalyzed by l-proline in the hydrophobic compartment of multicompartment micelle (MCM) nanoreactor consisting of amphiphilic bottlebrush copolymer, which minimizes the water content at the reactive site. Through performing dissipative particle dynamics (DPD) simulation, it is found that the “clover-like” morphology of the MCM is formed from multiblock copolymer with a sequence of ethylene oxide-based hydrophilic blocks, styrene lipophilic blocks, l-proline catalyst blocks, and a pentafluorostyrene fluorophilic block in aqueous media. We find that the vicinity of the catalyst in the clover-like MCM has a low dielectric environment, which could facilitate the aldol addition reaction. Our DFT calculations demonstrate that the asymmetric aldol addition of l-proline-catalyzed acetone and 4-nitrobenzaldehyde is energetically more favorable under the low dielectric environment in MCM compared with other commonly used solvents such as DMSO, water, and vacuum condition.

## 1 Introduction

The aldol addition reaction, discovered originally by Kane in 1838, stands as one of the fundamental reactions in organic synthesis, wherein the formation of a carbon–carbon (C–C) bond occurs as a result of the reaction.^1,2^ Despite the discovery of this asymmetric reaction more than a century ago, it continues to present challenges, particularly in achieving high yields and selectivity when conducted in water as a reaction medium.^3,4^ Water can frequently inhibit the activity of the catalyst or alter the enantioselectivity due to its interference with critical ionic interactions and hydrogen bonds that stabilize the transition states of the reactions.^5,6^ Consequently, catalytic asymmetric reactions that can be performed with water are of current interest as water is a desirable solvent regarding environmental concerns, safety, and cost.^7,8^ Hence, special design considerations are necessary for conducting asymmetric reactions in the water phase.

In this context, it is noted that the utilization of micelle as a nanoreactor has been intensively studied to achieve such a reaction design in the water phase.^9−12^ The self-assembled multicompartment micelles (MCMs), formed spontaneously by amphiphilic molecules in water solvent, possess significant potential for applications as selective storage media for guest molecules.^13−15^ In particular, the presence of a segregated hydrophobic core can establish a favorable microenvironment for encapsulating the guest molecules and facilitating the selective release of incompatible hydrophobic contents.^16,17^ Therefore, utilizing multicompartment micelles (MCMs) as a support system for catalysis seems to be an excellent promise in achieving the site isolation of catalytic moieties.^18,19^ Covalently attaching the catalyst to the hydrophobic blocks of the copolymer enables multicompartment micelles (MCMs) to establish a hydrophobic microenvironment, making them suitable for the desired reactions within an aqueous medium. This approach allows the effective confinement and protection of catalytic species within the MCMs, leading to enhanced catalytic performance and selectivity.^20^

Among the catalytic moieties, amino acid proline (l-proline) has first been reported as a widely utilized chiral organocatalyst facilitating asymmetric C–C bond formation.^10,21−24^ Notably, when present in minimal water quantities, l-proline (**Proline**) exhibits remarkable catalytic activity in promoting aldol addition reactions between ketones and aldehydes, leading to high yields and enantiomeric excesses at room temperature.^25^ In contrast, at high water concentrations or in the presence of water alone, an adverse effect is observed in the reactions, leading to low yields and a reduction in enantioselectivity.^11,25^ Additionally, water is often considered an unsuitable solvent for hydrophobic organic compounds.^26,27^ Therefore, conducting catalytic reactions in water requires the creation of a hydrophobic environment within an overall aqueous medium.

**Figure 1:**
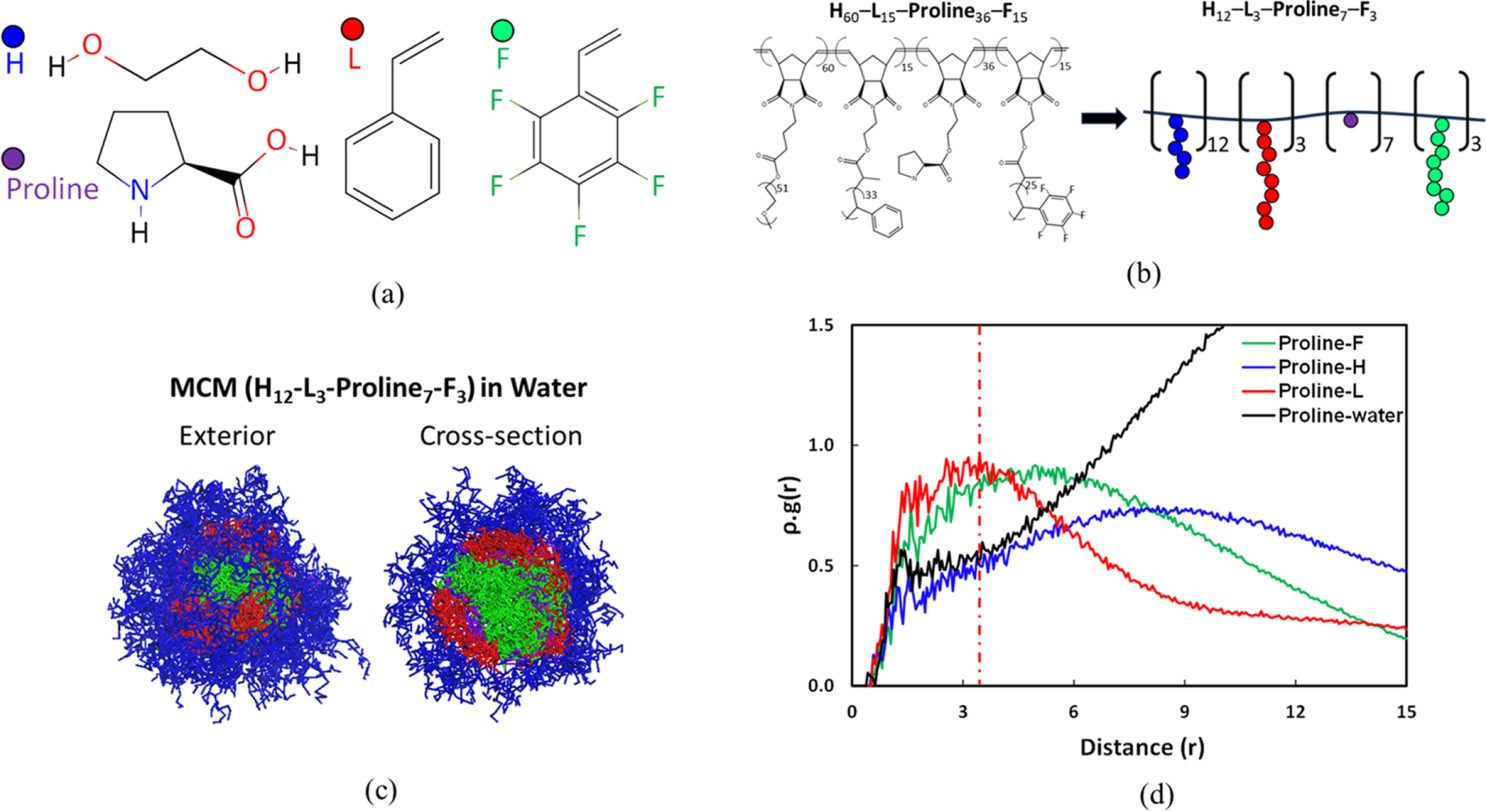
(a) Colored beads used as a visual representation of chemical structures (**H**, **L**, **F** blocks and **Proline** catalyst are represented by blue, red, green, and purple colors, respectively); (b) coarse-grained model of bottlebrush copolymer; (c) DPD simulation results for **MCM**; (d) pair correlation function analysis for the pairs of block **Proline** with blocks **H**, **L**, **F**, and water represented by blue, red, green, and gray lines, respectively.

Recently, we established the self-assembled MCM nanoreactor consisting of poly(norbornene)-based amphiphilic bottlebrush copolymers with **Proline** catalyst covalently attached adjacent to lipophilic (**L**) styrene or fluorophilic (**F**) pentafluorostryene blocks in water.^20^ In this **Proline**-catalyzed aldol addition study, surfactants and block copolymers created a hydrophobic core that guards the catalyst from an aqueous environment. This arrangement facilitates the reactivity of organic substrates within the aqueous system. As the molecular variables such as block sequence, block ratio, and catalyst location of the polymer play a pivotal role in determining the micelle morphology that impacts the catalytic activity, we considered these molecular variables in the design criteria.^28−32^ Among various types of micelles, we reported that “clover-like” **H-L-Proline-F** sequenced bottlebrush copolymers, illustrated in Figure 1a, exhibited the highest yield and selectivity due to the **Proline** catalyst adjacent to the **L** domain within the micelle. However, it should be noted that the underlying mechanism for such enhancement remains unclear as cryogenic transmission electron microscopy (cryo-TEM) images cannot provide detailed information about the local microstructures surrounding the catalyst. Compared with experimental endeavors, theoretical calculations and simulations have emerged as essential tools for unraveling the microscopic characteristics of micelles and providing detailed insights into their molecular-level structures.

In this study, we employed a multiscale modeling approach to thoroughly understand the reaction mechanisms in the self-assembled MCM, mainly focusing on the kinetics and transition-state structures. We first performed dissipative particle dynamics (DPD) simulations to investigate the morphology of MCM and the dielectric environment around the **Proline** catalysts within the MCM. Then, we performed density functional theory (DFT) calculations with an implicit solvation model to calculate the transition states for the aldol addition reaction of acetone (AT) and 4-nitrobenzaldehyde (NBA). Previously, Yang et al.^16^ have reported a direct aldol reaction via an enamine-mediated mechanism in dimethyl sulfoxide (DMSO), and Tafida et al.^33^ have reported an asymmetric aldol reaction in acetone medium. Both studies emphasize that the aldol reaction was assisted by solvents with a reduction of energy barriers. In this study, we aim to elucidate the reaction mechanism of the aldol addition reaction of acetone (AT) and 4-nitrobenzaldehyde (NBA) in a unique environment within the self-assembled MCM, which can provide direct information about the rate-determining step for reaction kinetics. We believe this study can shed light on the design guide of MCM to advance the kinetics of the aldol addition reaction in the water phase.

## 2 Modeling and Simulation Methods

### 2.1 DPD Simulation of Multicompartment Micelle

To characterize the internal morphology of the MCM, we performed DPD simulations. For DPD simulation, we calculated the Flory–Huggins χ_AB_-parameter for molecular A–B pairs such as polymer–water, water–water, and polymer–polymer pairs, from the mixing energy Δ*E*_AB_^mix^ by eq 1
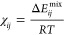
1In the previous study,^29,34^ we developed a computational method to estimate the χ-parameter consistently and accurately using the improved mixing energy as defined by eq 2
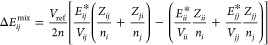
2where *Z*_*ij*_, *V*_*ij*_, *n*, *V*_ref_, and *E*_*ij*_ refer to the coordination number of the molecule *j* around the molecule *i*, the volume enclosed by the Connolly surface over the combined pair of molecules *i* and *j*, the number of monomeric units, reference volume, and interaction energy between molecules *i* and *j*, respectively. Since the Δ*E*_*ij*_^mix^ in eq 2 utilizes more molecular information compared with the original form of mixing energy, , the χ-parameter values were in good agreement with the experimental values.^34^

To calculate molecular information for eq 2, we prepared molecular models using DFT calculation with B3LYP functional and 6-31G(d,p) basis set in Jaguar.^35^ The Connolly volume, interaction energy, and the coordination numbers of polymer–water, water–water, and polymer–polymer pairs were obtained using the modeling software *Materials Studio*(36) to calculate the mixing energy Δ*E*_AB_^mix^.

For DPD simulation, the bead–spring model was employed, and the motions of beads are described by integrating Newton’s equations of motion as follows^37^^37^
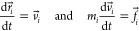
3where *r⃗*_*i*_, *v⃗*_*i*_, and *m*_*i*_ denote the position, velocity, and mass of the *i*th particle, respectively, and *f⃗*_*i*_ denotes the force acting on the *i*th particle. The force acting on a bead contains three parts
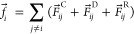
4where *F⃗*_*ij*_^C^, *F⃗*_*ij*_^D^, and *F⃗*_*ij*_^R^ denote conservative repulsive, dissipative, and random force depending on the position and velocity of the *i*th particle, respectively. The origin of physics and detailed methodology for DPD simulation can be found in the literature.^38−43^ Relevant computational conditions in our DPD simulation of MCM can be found in previous publications.^20,28−30,32,34,44−46^

In our DPD simulations, the simulated systems were set to have 5% polymer (**H**_**60**_**-L**_**15**_**-Proline**_**36**_**-F**_**15**_ in Figure 1b) and 95% water. Although this polymer concentration is higher than that used in experiments, a high concentration is employed in our simulations to ensure more intensive polymer–polymer interactions and thereby to better accomplish the self-assembly process during DPD simulation.^12,20,28,30^ The cubic simulation box size was set as 30 × 30 × 30 reduced DPD length units (*r*_c_^3^) with a grid spacing of 1.0 and a bead density of 3.0 (*r*_c_^3^/*V*_m_) to use the linear relationship between the repulsion parameter *a*_*ij*_ and the corresponding Flory–Huggins χ_*ij*_ parameter, as derived by Groot and Warren.^41^

### 2.2 DFT Modeling for Proline-Catalyzed Aldol Addition Reaction

To investigate the **Proline**-catalyzed aldol condensation of acetone (AT) and 4-nitrobenzaldehyde (NBA) in MCM (Figure 1c), we implemented DFT calculation through *Jaguar* package^35^ using B3LYP functional^47^ and 6-31G(d,p) basis set with Poisson–Boltzmann implicit solvation model^48−50^ to consider local molecular environment in the vicinity of reaction site within the multicompartment micelle. For the Poisson–Boltzmann implicit solvation model, we employed the dielectric constant (ε) of 80.37 and 47.24 for water and DMSO with probe radius values of 1.40 and 2.41 Å, respectively. Transition states were obtained from the quadratic synchronous transit (QST) search method,^51^ and the analytical frequency calculations were performed to ensure the transition states have exactly one imaginary frequency. Additionally, intramolecular reaction coordinate (IRC) analysis was performed to indicate that the resulting transition state can serve as a pathway leading from the reactants to the products.

## 3 Results and Discussion

### 3.1 Structural Analysis of MCM for Dielectric Environment around Catalyst

First, we prepared the MCM-based nanoreactor consisting of **H**_**60**_**-L**_**15**_**-Proline**_**36**_**-F**_**15**_ using DPD simulation. For this, we calculated Flory–Huggins χ-parameters using eqs 1 and 2.^29,34^ As summarized in Table 1, we found that, among the amphiphilic blocks, pentafluorostyrene–water (**F–W**) pair exhibits the highest χ-parameter value (χ_F–W_ = 0.963), followed by styrene–water (**L–W**) pair (χ_L–W_ = 0.756), l-proline–water (**Proline–W**) pair (χ_P–W_ = 0.459), and ethylene oxide–water (**H–W**) pair (χ_H–W_ = 0.197), implying that blocks **F** and **H** are expected to be the most hydrophobic and hydrophilic, respectively. Please note that, in our coarse-grained modeling approach, we have scaled down the polymer chain **H**_**60**_**-L**_**15**_**-Proline**_**36**_**-F**_**15**_ to **H**_**12**_**-L**_**3**_**-Proline**_**7**_**-F**_**3**_ to improve the efficiency of DPD simulations. Additionally, we have further scaled down the number of units in each side chain by considering the reference molecular volume of the l-proline catalyst. This scaling approach allows us to achieve computational efficiency while preserving essential characteristics of the system.

**Table 1:** Calculated Flory–Huggins χ-Parameters for Molecular Pairs

**χ**_**A-B**_	**L** lipophilic styrene	**H** hydrophilic ethylene oxide	**F** fluorophilic pentafluorostyrene	**Proline**l-proline	**W** water
**L**	0	1.462	0.945	1.075	0.756
**H**	1.462	0	0.735	0.531	0.197
**F**	0.945	0.735	0	1.220	0.963
**Proline**	1.075	0.531	1.220	0	0.459
**Water**	0.756	0.197	0.963	0.459	0

The amphiphilic bottlebrush block copolymers (**H**_**12**_**-L**_**3**_**-Proline**_**7**_**-F**_**3**_) self-assemble in water to form a multicompartment micelle with hydrophilic blocks (**H**) forming the outermost shell directly in contact with water molecules and hydrophobic blocks (**L** and **F)** forming the inner structures, such as the core of the micelle. Regarding molecular interactions, distinct phase separation of block **H** from block **L** in the micelle is expected since the highest χ-parameter (χ_H–L_ = 1.462) in Table 1 indicates that blocks **H** and **L** are immiscible. Likewise, blocks **L** and **F** are highly prone to phase separation within the core of the micelle due to their relatively high χ_L–F_ value of 0.945. Block **Proline** is less hydrophilic than block **H** (χ_P–W_ > χ_H–W_). At the same time, block **Proline** is not readily miscible with blocks **L** and **F**, indicating that block **Proline** may be sandwiched between the core (**L** and **F**) and shell (**H**) within the micelle.

We conducted DPD simulations to predict the morphologies of **MCM** using the χ-parameters in Table 1. We observe a distinct phase separation in the core region between blocks **L** and **F**, which is consistent with the immiscibility of blocks **L** and **F** as confirmed by the large χ_*L*–*F*_ value (0.945). **MCM** yields core–shell morphology as the block **L** domain covers block **F** domains. To further quantitatively characterize the structure of **MCM**, we implemented the pair correlation function (ρ_*i*_*g*(*r*)) for the pairs between **Proline** and other blocks using the following equation
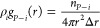
5where ρ_*i*_ denotes the number density of block *i* such as blocks **H**, **L**, and **F**, and *r* and Δ*r* denote the distance between **Proline** and block *i* and the shell thickness, respectively.

From Figure 1d, we found that ρ_L_*g*_Proline-L_ (*r*) with red color and ρ_F_*g*_Proline-F_ (*r*) with green color exhibit stronger intensities than other pair correlations such as ρ_W_*g*_Proline-W_ (*r*) with black color and ρ_H_*g*_Proline-H_ (*r*) with blue color up to *r* = ∼5.5, indicating that block **Proline** is located in the vicinity of blocks **L** and **F**. Particularly, ρ_L_*g*_Proline-L_ (*r*) exhibits slightly stronger intensity than ρ_F_*g*_Proline-F_ (*r*) up to *r* = ∼ 4.5, which is consistent with the χ-parameters in Table 1. Please note that the ρ_*i*_*g*(*r*) information is useful to estimate the local environment around the block **Proline** within MCM.

Since the aldol reaction takes place at block **Proline**, the local environment of **Proline** will affect the reaction as a dielectric environment. To perform DFT calculations investigating homogeneous catalytic process, we estimated the dielectric constant of this local environment of **Proline** by summing up the contribution of each block linearly using eq 6
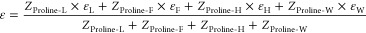
6where Z_**Proline-*****i***_ and ε_*i*_ denote the coordination number and dielectric constant for block *i* such as **L**, **F**, **H**, and **W**, respectively. Z_**Proline-*****i***_ is calculated by integrating the ρ_*i*_*g*(*r*) in eq 5 up to *r* = 3.45, where the position of the first peak in the **Proline**-**L** pair is identified as the highest among all **Proline***-***i** pairs and is then used to determine the number of beads in the first coordination sphere. Consequently, the dielectric constant (ε) estimated by eq 6 is 20.38, which is used to calculate the reaction energies and barriers in homogeneous catalysis through DFT calculation (Table 2).

**Table 2:** Summary of Dielectric Constant (***ε***) of Each Molecule (***i***) and the Coordination Number (**Z**_**proline–*****i*****)**_ of Proline and *i*

molecule (*i*)	dielectric constant (*ε*)	coordination number (*Z*_proline–*i*_)
**L**	2.55	1.65
**H**	13.53	0.86
**F**	1.86	1.36
**Water**	78.40	1.04

### 3.2 DFT Modeling of Aldol Addition of Acetone and 4-Nitrobenzaldehyde in MCM

**Scheme 1:**
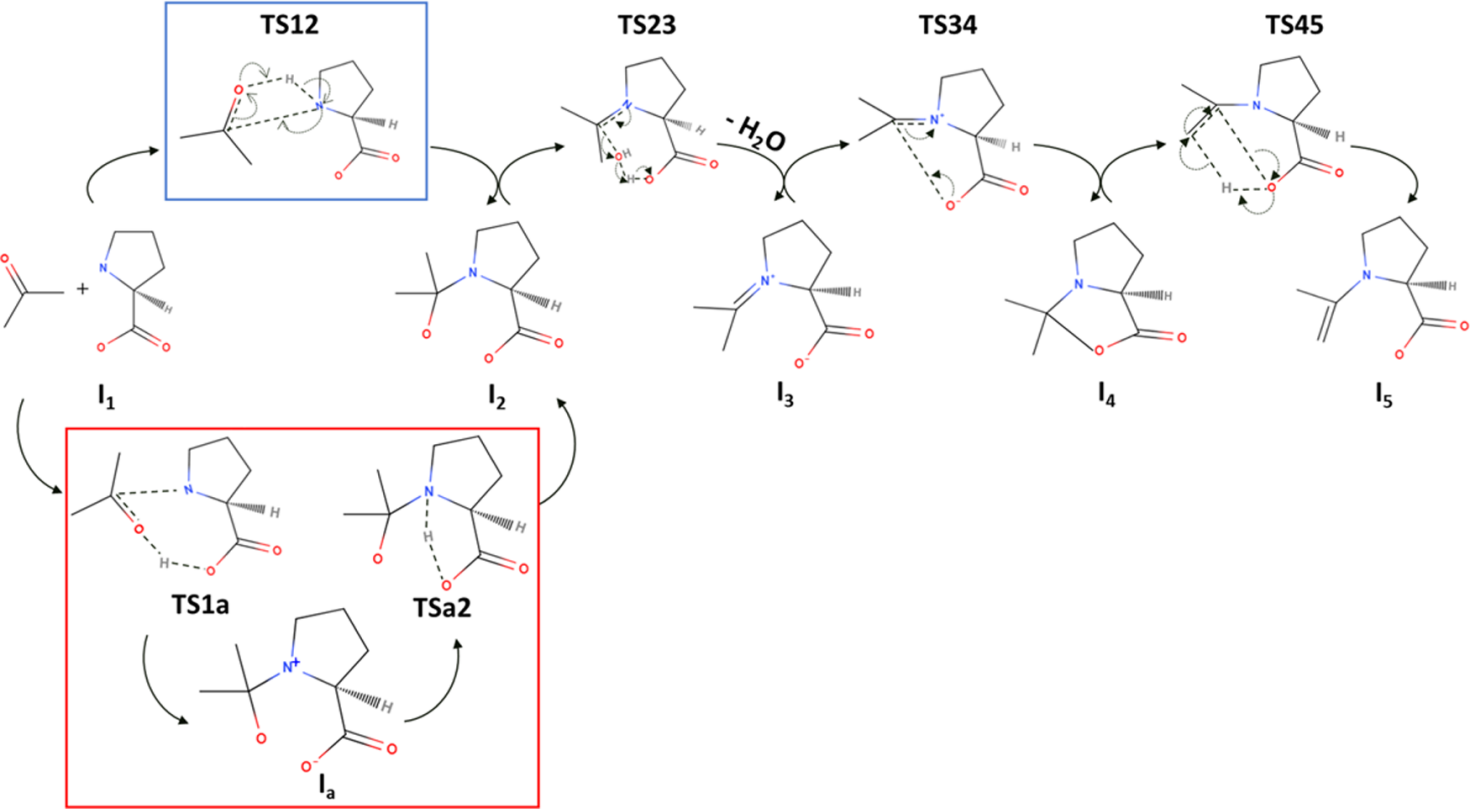
Proposed Mechanism of the Proline-Catalyzed Asymmetric Aldol Reaction of Acetone via One-Step (Blue Box) and Two-Step (Red Box) Protonation Mechanisms

**Scheme 2:**
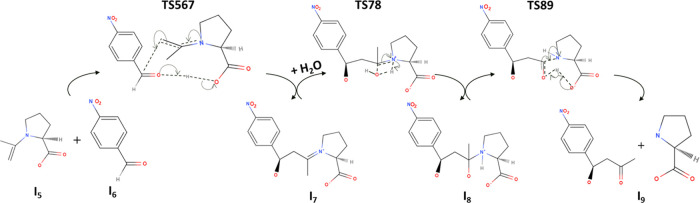
Proposed Mechanism of the Proline-Catalyzed Asymmetric Aldol Reaction of Acetone and 4-Nitrobenzaldehyde

We investigated the asymmetric aldol addition between AT and NBA using the DFT method within the MCM obtained from DPD simulation. In the previous studies,^16,33^ the l-proline-catalyzed aldol reaction mechanisms have been reported to have two stages: first, l-proline catalysts react with acetone to enamine as depicted in Scheme 1; subsequently, C–C bond connection is formed upon the addition of NBA as depicted in Scheme 2.

**Figure 2:**
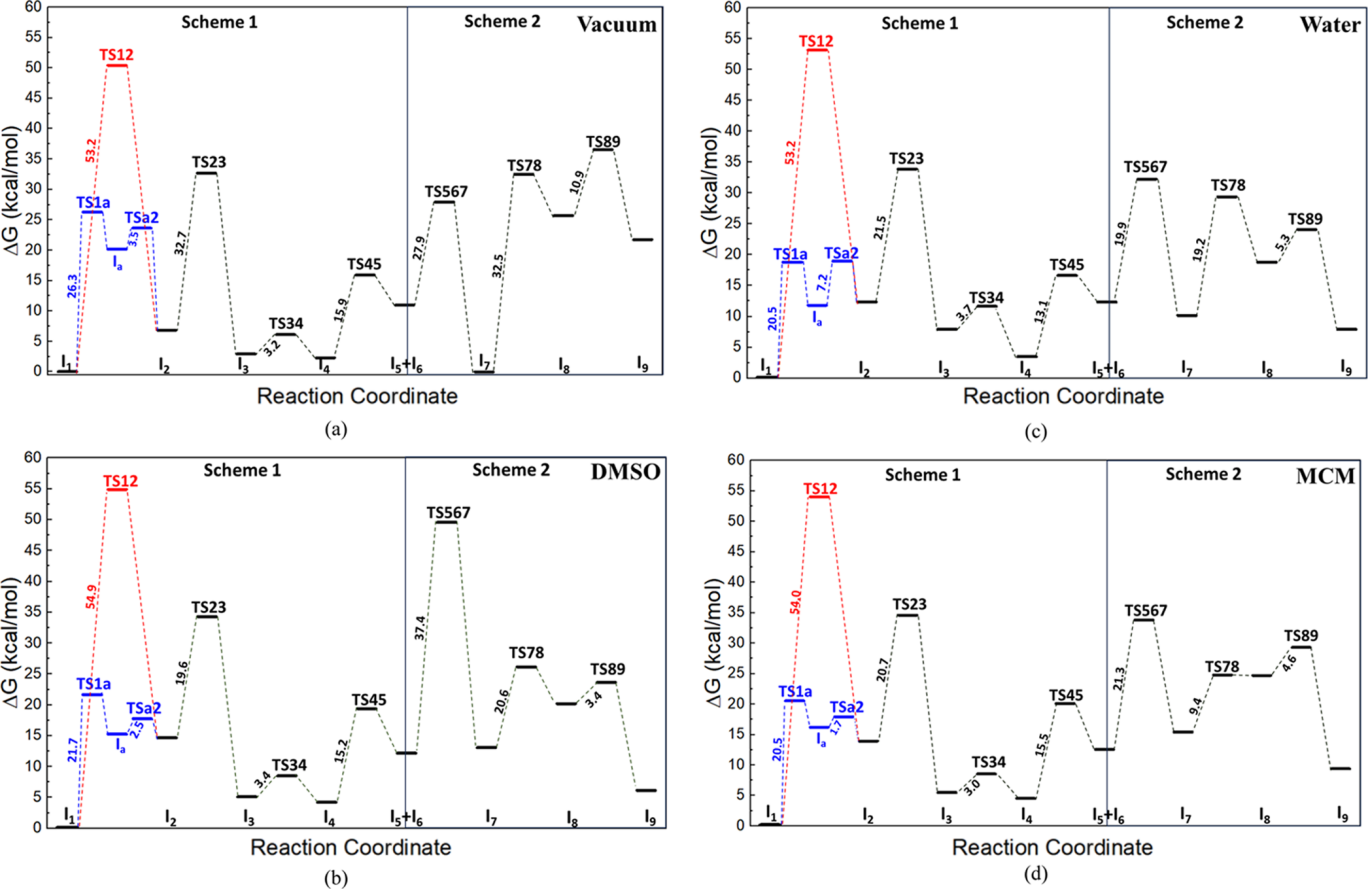
Free energy profiles with activation energies as a function of the reaction coordinates for the l-proline-catalyzed aldol reaction between acetone and 4-nitrobenzaldehyde under (a) vacuum, (b) DMSO, (c) water, and (d) MCM environments. The red and blue bars correspond to the one-step and two-step protonation mechanisms, respectively.

Figure 2a–d presents the free energy profiles of the aldol reaction as a function of reaction coordinate, which displays the energy barriers of all of the elementary reaction steps under the different solvent environments, including water, DMSO, vacuum, and MCM. The energy change of the overall reaction (Δ*E*_**net_solvent**_) required to convert reactants **I**_**1**_ (AT and NBA) into **I**_**9**_ is found to be smallest when using water as the solvent (Δ*E*_**net_water**_ = 12.6 kcal/mol). This is followed by MCM (Δ*E*_**net_MCM**_ = 13.4 kcal/mol) and then DMSO (Δ*E*_**net_DMSO**_ = 13.6 kcal/mol). Although the variation in the net reaction energy across different solvent environments is not substantial, the rate of the chemical reaction is predominantly determined by the activation energies of individual elementary steps. This relationship is elucidated by the Arrhenius equation. Hence, accurately predicting the reaction rate necessitates a thorough consideration of the activation energies associated with each elementary step.

Scheme 1**-TS12**. The first reaction, often known as the rate-determining step in the aldol addition reaction between AT and NBA, where acetone reacts with the l-proline catalyst, denoted as **I**_**1**_ at 0.0 kcal/mol, becomes hemiaminal intermediate **I**_**2**_ in Scheme 1. The asymmetric aldol reaction starts with the nucleophilic attack of the N atom in the l-proline catalyst to the C atom of the carbonyl group in AT as LUMO and HOMO are reported to be localized on the C atom of carbonyl in AT and the N atom of l-proline catalyst, respectively, and thus forming a C–N bond.^33^ At the same time, there is proton transfer occurring from AT to l-proline.

Herein, we consider the two proton transfer mechanisms. One, a proton is transferred between the carbonyl oxygen of acetone and the amine group of l-proline via the strained four-membered cyclic transition state **TS12** in one step as proposed by Boyd et al.^52^ On the other hand, the oxygen atom of the ketone group in AT attacks the hydrogen atom of the hydroxyl group in l-proline (denoted as **TS1a**), resulting in the formation of a zwitterionic intermediate state **I**_**a**_.

The next step involves an additional intramolecular proton transfer from N^+^ to O^–^ (**TSa2**). Regardless of solvent environments, our calculation results indicate that the two-step proton transfer mechanism is energetically much more favorable for the formation of hemiaminal intermediate **I**_**2**_ than the one-step proton mechanism. Under DMSO, the activation energy (*E*_**a**_) of **TS1a** is 21.7 kcal/mol, whereas **TS12** requires 54.9 kcal/mol to proceed further. This aligns with a previous report showing a lower energy barrier for **TS1a** (*E*_**a**_ = 95 kJ/mol, equivalent to 22.7 kcal/mol) compared with **TS12** (*E*_**a**_ = 179 kJ/mol, equivalent to 42.8 kcal/mol).^16^ Likewise, Under the MCM environment, **TS12** exhibits the lowest *E*_**a**_ among different solvent environments with *E*_**a**_ = 54.0 kcal/mol, and **TS1a** has a lower *E*_**a**_ than **TS12** with 20.4 kcal/mol. The same trend is observed under the water solvent. In general, we found that **TS1a** exhibits a lower energy barrier than **TS12**, and **TS1a** under water solvent shows the lowest *E*_**a**_ compared with other solvents, which contradicts the experimental results.^53−56^

**Figure 3:**
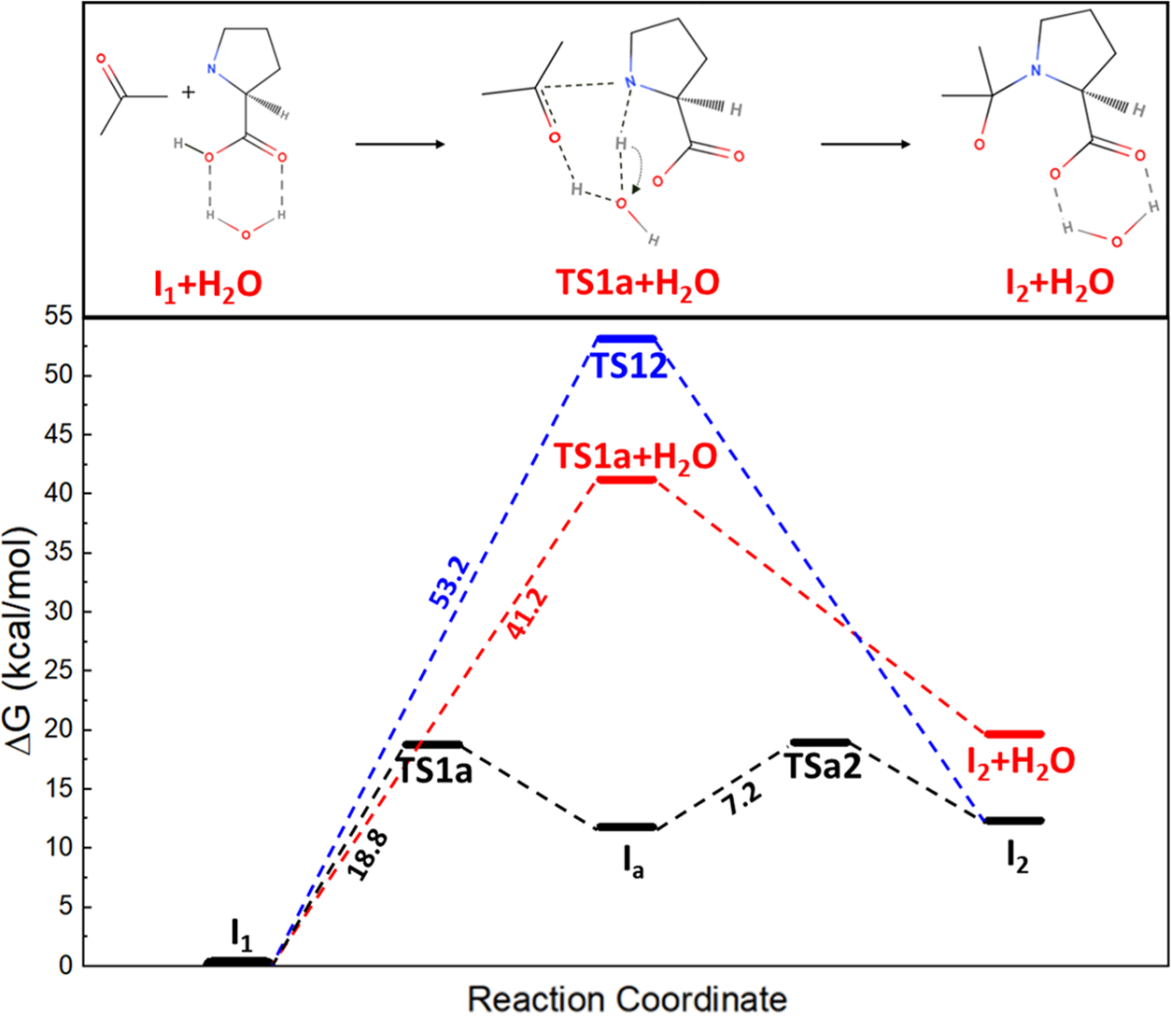
Reaction pathway of hemiaminal intermediate I_2_ formation from **I**_**1**_ under the presence of a water molecule and the corresponding reaction and kinetic energies are shown in the red bar. The blue and black bars represent one-step and two-step mechanisms, respectively.

Therefore, it is essential to explicitly consider water molecules to properly address the hindrance of water in the aldol addition reaction. The presence of water molecules surrounding the AT and l-proline catalysts can significantly alter the reaction mechanism. As proposed by many theoretical studies, the involvement of water introduces new reaction pathways as water serves not only as a solvating agent but also as a proton transfer assistant, and thus influencing the overall reaction pathways and kinetics.^57−60^ To evaluate the dual behaviors of water, we calculated the reaction kinetics in the presence of a single water molecule, as depicted in Figure 3. Unlike **TS12** and **TS1a**, water in **TS1a+H_2_O** allows for proton transfer to the carbonyl oxygen of acetone, which requires *E*_a_ = 41.2 kcal/mol for the reaction to proceed. Although *E*_a_ for **TS1a+H_2_O** is higher than **TS1a** under the implicit water solvent, considering that the aldol addition is the least energetically favorable in the water solvent experimentally, **I_2_** formation via **TS1a+H_2_O** may be more reasonable.

Scheme 1**-TS23, T34, T45, and T35**. As hemiaminal intermediate completely forms in **I**_**2**_, the O–H bond of l-proline breaks to remove a water molecule to form a zwitterionic iminium ion **I**_**3**_, which is an exothermic reaction regardless of solvents, and our calculation predicts the substantially higher exothermicity in DMSO medium with Δ*E*_**23_DMSO**_ = −9.6 kcal/mol, followed by MCM with Δ*E*_**23_MCM**_ = −8.4 kcal/mol, water with Δ*E*_**23_water**_ = −4.4 kcal/mol, and vacuum with Δ*E*_**23_vacuum**_ = −3.9 kcal/mol. Although the reaction energy Δ*E*_**23**_ is the most exothermic in DMSO, its *E*_**a**_ for **TS23** is 19.4 kcal/mol, which is twice larger than *E*_***a***_ of water (8.8 kcal/mol). This result indicates that removing water is most feasible in a water solvent.

There are two possible reaction pathways for the iminium-to-enamine transformation (reactions from **I**_**3**_ to **I**_**5**_ via **I**_**4**_). One possible reaction path is the two-step mechanism in which the reaction is initiated with the attack of the O^–^ atom to the C atom from the C–O bond to form **I**_**4**_ via **TS34**, followed by the dissociation of the C–O bond **(TS45)** to form enamine **I**_**5**_. According to the free energy diagram, the transition state of the endothermic reaction **I**_**4**_ to **I**_**5**_, denoted as **TS45**, generally exhibits higher activation barriers than **TS34**, indicating the C–O bond activation requires higher energy than the attack of O^–^ to carbon in the imine group. For example, **TS34** is the lowest under the MCM environment with *E*_**a**_ = 3.0 kcal/mol, and **TS45** is significantly higher with *E*_**a**_ = 15.5 kcal/mol.

**Figure 4:**
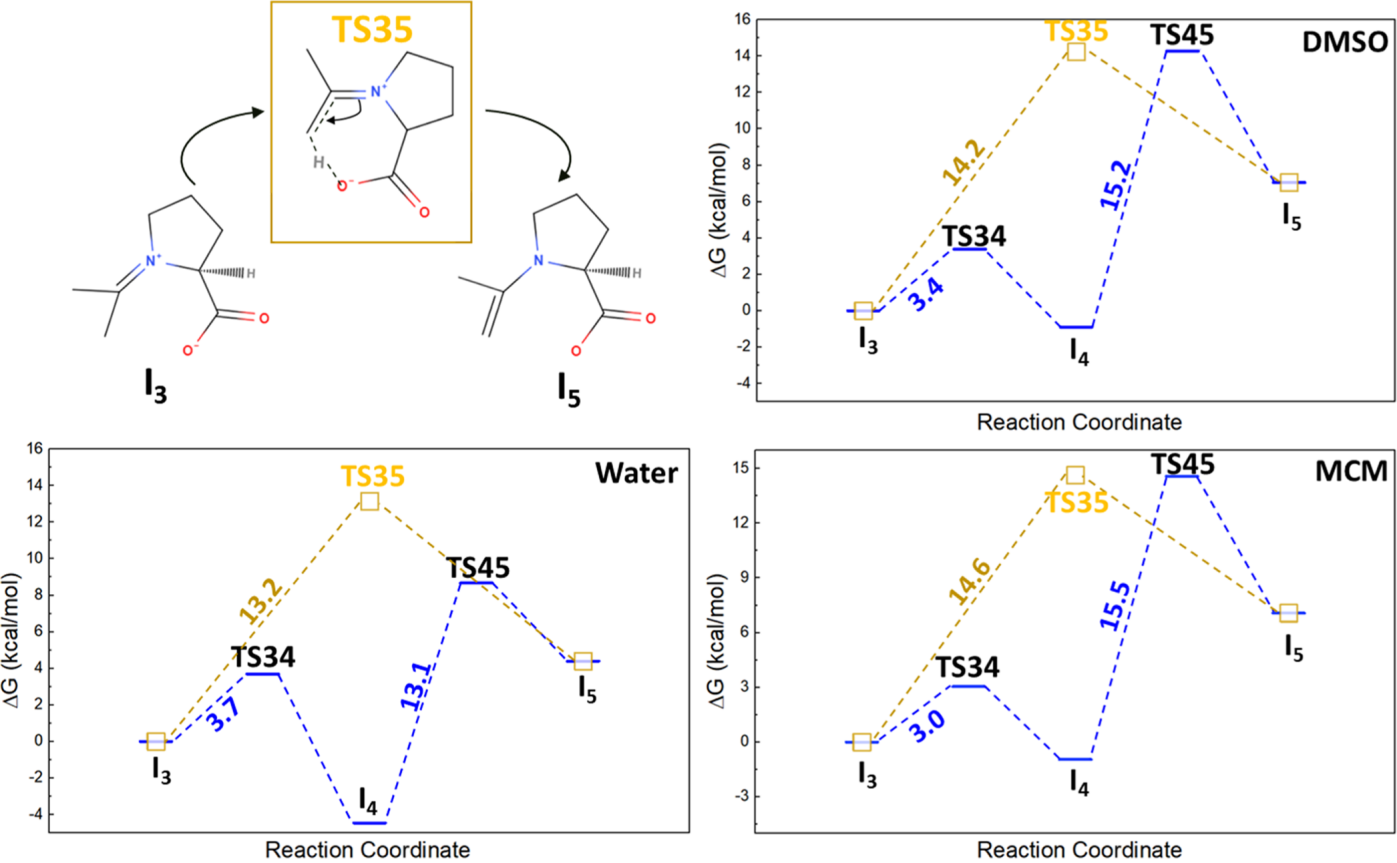
Schematic illustration of a single-step transformation mechanism from iminium to enamine, **I**_**3**_ to **I**_**5**_ via **TS35**, and the free energy diagram of a single-step (yellow square) and a two-step mechanism via **TS34**, **I**_**4**_, and **TS45** (blue bar) under DMSO, water, and MCM environments.

A similar trend is observed in other dielectric environments. Alternatively, as illustrated in Figure 4, the O^–^ atom can directly attack the hydrogen in the methyl group to form **I**_**5**_ in a single step via **TS35** as initially suggested by List and co-workers.^22^ We compared the energy barriers of a single-step transition state **TS35** with **TS45**, which is the rate-determining step of the two-step iminium-to-enamine transformation. Even though the positions of **TS35** and **TS45** in the free energy profiles may seem similar for DMSO and MCM, the *E*_**a**_ of **TS35** is slightly smaller than the *E*_**a**_ of **TS45**. For example, the *E*_**a**_ of **TS45** under the MCM environment is 15.5 kcal/mol, whereas the *E*_**a**_ value of **TS35** is 14.6 kcal/mol.

While the *E*_***a***_ difference may appear marginal at room temperature, the single-step transformation becomes more energetically favorable, resulting in a faster reaction rate as the temperature increases, irrespective of the solvent environment. In water, however, the two-step mechanism is preferred over the single-step mechanism due to the highly exothermic nature of **I**_**4**_ formation from **I**_**3**_, leading to **TS45** lying lower than **TS35** in the free energy diagram. To sum up, **Proline**-catalyzed asymmetric aldol addition of acetone to enamine is the fastest under the MCM environment as the highest activation barrier **TS23** among the reaction pathways from **I**_**1**_ to **I**_**5**_ is the lowest compared with DMSO, water, and vacuum conditions.

Scheme 2**-T567, TS78, and TS89**. As proposed in Scheme 2, once the enamine is formed, the C = C double bond of enamine reacts with the C atom of carbonyl in **NBA** to form a C–C bond, and the proton transfer from the oxygen of the aldehyde group to the oxygen of carboxylic acid as seen in the intermediate **I**_**7**_. To proceed with this reaction, the energy barrier required to overcome **TS567** is the lowest under water with 19.9 kcal/mol, followed by MCM with 21.3 kcal/mol, vacuum with 27.9 kcal/mol, and DMSO with 37.4 kcal/mol. While the Δ*E*_**567**_ remains exothermic under water and vacuum, it is endothermic for MCM and DMSO with Δ*E*_**567_MCM**_ = 2.9 kcal/mol and Δ*E*_**567_DMSO**_ = 0.9 kcal/mol, respectively. It is noted that the *E*_**a**_ for **TS567** under the MCM environment (*E*_***a***_ = 21.3 kcal/mol) is marginally higher than that for **TS1a** (*E*_**a**_ = 20.5 kcal/mol), indicating that **TS567** is potentially the rate-determining step. Likewise, *E*_**a**_ for **TS567** under the DMSO solvent is higher than **TS1a**. It can also be inferred that the reactions involving the nucleophilic attack at the carbonyl carbon group, such as **TS12** and **TS567**, typically require a lot of energy to overcome the barrier compared with reactions involving proton transfers.

Next, reactions from intermediate state **I**_**7**_ to final product **I**_**9**_ include the cleavage of the C–N^+^ bond by adding a water molecule for the hydrolysis. As depicted in **TS78**, the oxygen of water attacks the carbon atom of the imine complex, and at the same time, the O–H bond of water breaks apart to form the zwitterionic intermediate state **I**_**8**_. This water-assisted reaction energy, Δ*E*_**78**_, one of the most endothermic reactions, is the most sluggish in the water medium as *E*_***a***_ for **TS78** is the highest with *E*_**a**_ = 19.2 kcal/mol in the water, followed by DMSO (*E*_**a**_ = 13.1 kcal/mol) and MCM (*E*_**a**_ = 9.4 kcal/mol). Finally, the zwitterionic intermediate state **I**_**8**_ is split into l-proline and the aldol product, **I**_**9**_, through the scission of the C–N^+^ bond and the formation of a C=O double bond via a proton transfer from the O^–^ atom. This exothermic reaction needs relatively small energy barriers (as small as 3.4 kcal/mol under DMSO) compared with the previous transition states.

Overall, the reaction energy for l-proline-catalyzed aldol addition of AT and NBA is calculated to be similar for water, DMSO, and MCM. However, the kinetic energy exhibits a significant difference based on the solvent environment, which impacts the reaction rate. Our calculation indicates that the rate-determining step in the MCM has the lowest energy barrier (21.3 kcal/mol), followed by those of DMSO (37.4 kcal/mol) and water (41.2 kcal/mol). These results suggest that the MCM nanoreactor facilitates the aldol addition reaction by providing a local hydrophobic environment that reduces the water concentration around the reactant species and catalyst, enhancing enantioselectivity.

## 4 Conclusions

The aldol addition reaction, an essential reaction in organic chemistry, has often been reported to exhibit poor yields and low enantioselectivities when excessive water is present. However, we can utilize multicompartment micelle as a nanoreactor to facilitate the aldol reaction by providing a proper local hydrophobic environment around l-proline. In this study, we investigated probable mechanisms of the aldol reaction catalyzed by l-proline of block proline within the hydrophobic core of MCM using multiscale modeling.

Amphiphilic bottlebrush copolymers were self-assembled to form an MCM with a “clover-like” morphology. The amphiphilic bottlebrush copolymer comprises an ethylene glycol-based hydrophilic block, a styrene-based lipophilic block, an l-proline-attached block, and a pentafluorostyrene-based fluorophilic block in a sequence along the chain copolymer. From the internal structure of the MCM, the effective dielectric constant was estimated by summing the dielectric constant of each component linearly, which was considered for the implicit solvent media in our DFT calculations.

Through this study, we demonstrated that the l-proline-catalyzed asymmetric aldol addition reaction of acetone and 4-nitrobenzaldehyde is energetically more favorable in the micelle with the lowest energy barrier (21.3 kcal/mol) for the rate-determining step in comparison to other environments such as DMSO (37.4 kcal/mol) and vacuum (32.7 kcal/mol). In the case of the reaction in the water solvent, it is crucial to consider an explicit water molecule around acetone and the l-proline catalyst, as the reaction from **I_1_** to **I_2_** via **TS1a+H_2_O** becomes the rate-limiting step, with a corresponding energy barrier of 41.2 kcal/mol, the highest among other solvents.

Our finding indicates that the MCM nanoreactor facilitates the aldol addition reaction by creating a local hydrophobic environment that reduces the water concentration around the reactant species and catalyst, thus enhancing enantioselectivity.

## Supporting Information Available

Free energy profiles for every transition state are elucidated in this study (PDF)
