# Supplementary material for: Multiscale Modeling Approach for the Aldol Addition Reaction in Multicompartment Micelle-Based Nanoreactor
Source: J Phys Chem B. 2023 Nov 13;127(46):10067–76. doi: 10.1021/acs.jpcb.3c05858 (PMC10683011; doi:10.1021/acs.jpcb.3c05858)
Supplement: Supplementary file 1 — jp3c05858_si_001.pdf [file jp3c05858_si_001.pdf]

Supporting Information

Multiscale Modeling Approach for Aldol Addition Reaction in Multicompartment Micelle-based Nano-Reactor

Jinwon Cho,[1] Marcus Weck,[2] Sungu Hwang,[3,*] and Seung Soon Jang[1,*]

1 Computational NanoBio Technology Laboratory, School of Materials Science and Engineering, Georgia Institute of Technology, 771 Ferst Drive NW, Atlanta, GA 30332-0245, USA
2 Molecular Design Institute and Department of Chemistry, New York University, New York, NY, 10003, United States
3 Department of Nanomechatronics Engineering, Pusan National University, Miryang 50463, Korea

* Corresponding author:

Sungu Hwang sungu@pusan.ac.kr orcid.org/0000-0002-8722-9312
Seung Soon Jang seungsoon.jang@mse.gatech.edu orcid.org/0000-0002-1920-421X

Keywords: Multicompartment Micelle, Nano-Reactor, Aldol Reaction, Density Functional Theory, Homogeneous Catalysis

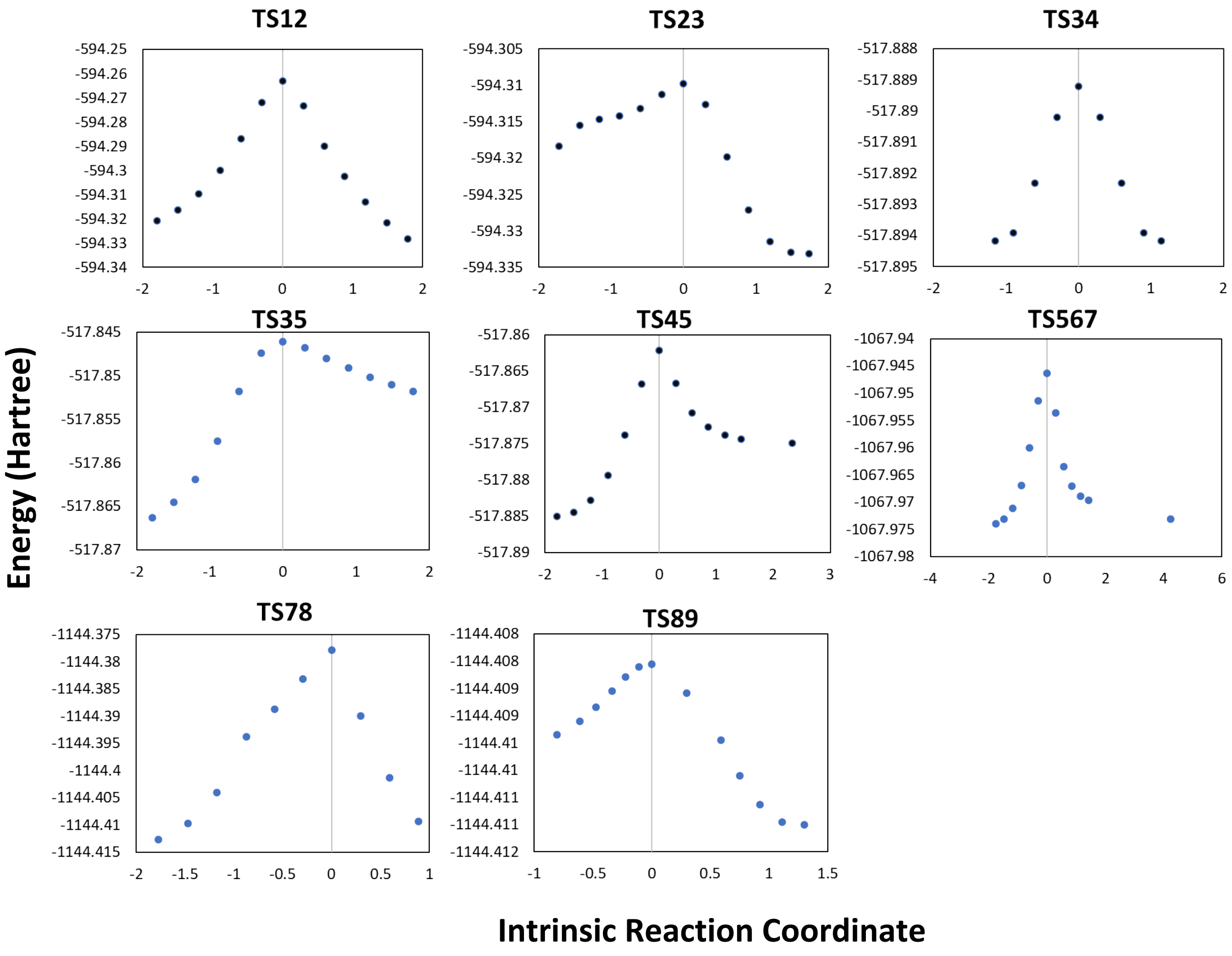

Figure S1. Free energy profile as a function of intrinsic reaction coordinate for each transition state used in this study.
